# American Medical Association

**Published:** 1873-04-01

**Authors:** W. B. Atkinson


### Miscellaneous.

### AMERICAN MEDICAL ASSOCIATION.

*Office of Permanent Secretary, Wm. B. Atkinson, M.D.*, 1400 *Pine street, cor. Broad, Philadelphia.*

The Twenty-fourth Annual Session will be held in St. Louis, Mo., May 6th, 1873, at 11 A.M.

Physicians desiring to present papers before the Association should observe the following rule:

"Papers appropriate to the several sections, in order to secure consideration and action, must be sent to the Secretary of the appropriate section at least one month before the meeting which is to act upon them. It shall be the duty of the Secretary to whom such papers are sent, to examine them with care, and, with the advice of the Chairman of his Section, to determine the time and order of their presentation, and give due notice of the same."

OFFICERS OF SECTIONS.

Chemistry and Materia Medica—Drs. R. E. Rogers, Philadelphia, Pa., Chairman; Ephraim Cutter, Boston, Mass., Secretary.

Practice of Medicine and Obstetrics—Drs. D. A. O'Donnell, Baltimore, Md., Chairman; Benjamin F. Dawson, New York, N. Y., Secretary.

Surgery and Anatomy—Drs. Edward Warren, Baltimore, Md., Chairman; W. F. Peck, Davenport, Iowa, Secretary.

Meteorology and Epidemics—Drs. George Sutton, Aurora, Ind., Chairman; Elisha Harris, New York, N. Y., Secretary.

Medical Jurisprudence, Hygiene, and Physiology—Drs. S. C. Busey, Washington, D. C., Chairman; A. B. Arnold, Baltimore, Md., Secretary.

Psychology—Drs. Isaac Ray, Philadelphia, Pa., Chairman; John Curwen, Harrisburg, Pa., Secretary.

AMENDMENTS TO BE ACTED ON.—(TO CONSTITUTION.)

*Resolved*, That the United States Marine Hospital Service be placed in the same relative position in the American Medical Association as the Medical Departments of the United States Army and Navy.

And that, in paragraph 2 of the 2d section, after the words "army and navy," the words "and the United States Marine Hospital Service" be inserted.

(TO BY-LAWS.)

SEC. III.—*Standing Committees.*—That, instead of a report on Medical Education, on Medical Literature, and Climatology and Epidemic Diseases, there shall be annually delivered before the Association, at its general meetings, an address in Medicine, an address in Surgery, and an address in Midwifery, or the Diseases of Children, the lecturers to be appointed this year by the President; afterwards by the Committee on Nominations.

Also, in section 6, after the words "the chiefs of the bureaus of the army and navy," be inserted "and the supervising surgeon of the United States Marine Hospital Service."

Secretaries of all Medical organizations are requested to forward lists of their Delegates, as soon as elected, to the Permanent Secretary. W. B. ATKINSON.

